# Assessment of the Genetic Diversity of a Local Pig Breed Using Pedigree and SNP Data

**DOI:** 10.3390/genes12121972

**Published:** 2021-12-10

**Authors:** Emil Krupa, Nina Moravčíková, Zuzana Krupová, Eliška Žáková

**Affiliations:** 1 Institute of Animal Science, 104 00 Prague, Czech Republic; krupova.zuzana@vuzv.cz (Z.K.); zakova.eliska@vuzv.cz (E.Ž.); 2 Faculty of Agrobiology and Food Resources, Institute of Nutrition and Genomics, Slovak University of Agriculture, 949 76 Nitra, Slovakia; nina.moravcikova@uniag.sk

**Keywords:** Přeštice Black-Pied pig, pedigree, SNP, inbreeding, ROH

## Abstract

Herein, the genetic diversity of the local Přeštice Black-Pied pig breed was assessed by the simultaneous analysis of the pedigree and single nucleotide polymorphism (SNP) data. The information about sire line, dam, date of birth, sex, breeding line, and herd for 1971 individuals was considered in the pedigree analysis. The SNP analysis (*n* = 181) was performed using the Illumina PorcineSNP60 BeadChip kit. The quality of pedigree and SNPs and the inbreeding coefficients (F) and effective population size (N_e_) were evaluated. The correlations between inbreeding based on the runs of homozygosity (F_ROH_) and pedigree (F_PED_) were also calculated. The average F_PED_ for all animals was 3.44%, while the F_ROH_ varied from 10.81% for a minimum size of 1 Mbp to 3.98% for a minimum size of 16 Mbp. The average minor allele frequency was 0.28 ± 0.11. The observed and expected within breed heterozygosities were 0.38 ± 0.13 and 0.37 ± 0.12, respectively. The N_e_, obtained using both the data sources, reached values around 50 animals. Moderate correlation coefficients (0.49–0.54) were observed between F_PED_ and F_ROH_. It is necessary to make decisions that stabilize the inbreeding rate in the long-term using optimal contribution selection based on the available SNP data.

## 1. Introduction

**Figure 1:**
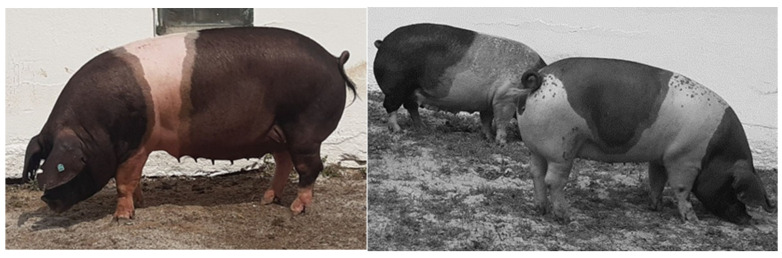
Přeštice Black-Pied sows.

The Přeštice Black-Pied (PBP) is a Czech local pig breed (Figure 1). At the end of the 19th century, the local pig breeds (mostly, Yorkshire, Sussex, Berkshire, and Essex British breeds) were crossed with boars imported into the Bohemia territory [1,2]. Subsequently, pig breeds including Wessex Saddleback (in 1952), Pietrain (in 1966), Welsh (in 1985), and Landrace (in 1986) were also used in crossing of the PBP breed [3]. However, during the WWII, the first bottleneck effect on genetic variation of pigs was observed after the ban on breeding black-spotted breeds of pigs was tightened even when their breeding was secretly maintained. Although the use of unlicensed boars and inbreeding contributed to the stability of the breed, there was an apparent impact on the genetic diversity of PBP pigs [1]. Since 1992, the PBP breed has been categorized as an Animal Genetic Resource (AnGR) and listed by the Food and Agriculture Organization of the United Nations (FAO) as threatened with extinction [4]. The PBP pig is recognized as a breed with a small body frame originally bred for good reproduction and fitness. It was reported that the litter size of the PBP pigs was 11.0 piglets. Among them, 10.3 piglets were born alive and 9.6 piglets were weaned. At 21 days of age, the litter weight was 54.4 kg [5]. In terms of meat production, the PBP pigs have showed, on average, 561 g of daily weight gain, 11.4 mm of backfat thickness, and 59.1% of lean meat content [6].

The biological diversity of the local pig breeds plays an important role in food security and nutrition. Nevertheless, it is seriously endangered by the continuous loss of genetic diversity [7]. Plentiful information is available regarding the genetic diversity of pigs, often provided by international cooperation [8]. It is important to determine the historical trend for small, closed, or isolated populations along with parameters that define genetic diversity parameters. This can be done only by detailed analysis of all available data about population under consideration. Only then, it is possible to make adequate management decisions to prevent the genetic diversity loss.

At present, two basic sources of data about population can be obtained. The pedigree data still represents the base for analyses of genetic diversity. In general, they have been collected over ten generations. However, the results obtained from pedigree data alone could misrepresent the extent of genetic diversity. This disadvantage could be addressed by the inclusion of population data from the SNP analyses. Single nucleotide polymorphism (SNP) analysis (preceded by the analysis of microsatellites) has contributed to a considerable refinement of the assessment of genetic diversity, especially in endangered animal populations. In PBP breed, assessment of genetic diversity based on microsatellites analysis [3] and pedigree data [9] has been studied. However, simultaneous analysis of the SNP and pedigree data of the PBP pig breed has not been performed till date.

The objective of the present study was to assess the genetic diversity of PBP pigs using the pedigree and SNP data.

## 2. Materials and Methods

### 2.1. Animals

The PBP breed belongs to the primitive indigenous breeds reared in semi-intensive or extensive production systems. This breed has been kept in situ in closed population for more than 25 years. It is characterized by a smaller body frame and mottled black and white coloration. The average weight of mature live boars and sows is 260–280 kg and 215–235 kg, respectively. Despite the fact that individuals are bred in herds with other commercial breeds, they do not have a strong selection pressure. In animals selected for breeding, the basic meat production and reproductive traits (listed in the Supplementary Table S1) are routinely determined and assessed. The database of Czech National program for Pig breeding (called CzePig) gathered and guided by the Czech Pig Breeders Association was used for determining the traits used for animal selection.

### 2.2. Pedigree Analysis

An active population of 324 dams and sires (animals used in breeding) in 2020 was considered the reference population for pedigree analysis of the PBP breed. Basic characteristics of the population are summarized in Table 1. The information about sire, dam, date of birth, sex, breeding line, and herd for 1971 individuals were considered for pedigree analysis. The pedigree of each individual in the reference population was tracked back to its earliest ancestor. The analyzed pedigree contains animals born between 1982 and 2020. Average generation interval (GI) was calculated as the average age of parents when their selected offspring was born (in years). The GI was computed for the four selection pathways (SS: sires to sons, SD: sires to daughters, DS: dams to sons, and DD: dams to daughters).

Inbreeding coefficient (F) of an individual was calculated using Wright’s coefficients and Meuwissen and Luo’s algorithm [10,11]. The differences of the inbreeding effect which were achieved during recent generations compared with inbreeding achieved during the more distant past were evaluated. Therefore, the total inbreeding coefficient of a given animal (F_AnT_) has been split into the “new” inbreeding coefficient (F_AnN_) and the “old” inbreeding coefficient (F_AnO_). The F_AnT_ was calculated as inbreeding coefficient of ancestors up to five generations back. In addition to the calculation of the inbreeding coefficient of an individual, the influence of inbreeding was also evaluated by the sires (F_SrN_, F_SrO_, and F_SrT_ as new, old, and total inbreeding, respectively), and dams (F_DmN_, F_DmO_, and F_DmT_ as new, old, and total inbreeding, respectively). Expected inbreeding coefficient was computed as the co-ancestry of the breeding animals assuming random mating [12]. The co-ancestry coefficient was obtained by applying Colleau’s algorithm [13].

Pedigree completeness index (PCI) was calculated to express the quality of the pedigree using the algorithm devised by MacCluer et al., which summarized the proportion of known ancestors in each ascending generation [14]. It quantifies the change in observed inbreeding in the pedigree [15]. The following equations were used:

$$I_{d}=\frac{4I_{d_{pat}}\;I_{d_{mat}}}{I_{d_{pat}}+I_{d_{mat}}}$$

And

$$I_{dk}=\frac{1}{d}\overset{d}{\underset{i=1}{\sum }}a_{i}$$

where *k* is the dam or sire line of an animal, *ai* is the proportion of known ancestors in generation *i*, and *d* is number of generations considered in the calculation of the pedigree completeness. For description of methods used to calculate maximum generations traced back, average complete equivalent generations, number of founders and number of ancestors explaining appropriate proportion of gene pool [16].

Different methods were used to compute the effective population size (N_e_). The N_e_ was calculated based on standard equation:

$$N_{e}=\frac{1}{2\;\Delta F}.$$

where ΔF is rate of inbreeding coefficient. Here, ΔFp is the ratio of the average inbreeding in an offspring and their direct parents and ΔFg was the ratio of the average inbreeding in an offspring to the average inbreeding in average parent’s generation. Both methods were described previously [12]. An algorithm devised by Pérez-Ensico was used for the calculation of N_e_-Ln and used the slope from logarithmic regression performed on year of birth of the PBP pigs in the pedigree analysis [17]. The approach defined by Gutierrez et al. [18], which uses the sum of the inbreeding coefficients of all individuals and their known ancestors, was used to compute the N_e_-Ecg. N_e_-Coan was computed using the additive genetic relationship against the inbreeding coefficient as described by Falconer and Mackay [12].

The basic pedigree data manipulations were performed using the R-project package [19]. The POPREP package v. 2.0, the CFC software v. 1.0, the PEDIG package, and the Endog software v. 4.5 were used for the pedigree analysis [20,21,22,23].

### 2.3. SNP Analysis

The genome-wide data were analyzed for 181 individuals (65 sows and 116 boars) born between 2005 and 2020. Bristles, frozen blood samples, and used insemination doses were the sources of DNA. All DNA analyses were performed at the laboratory of genetics of the Czech-Moravian Breeders’ Corporation. DNA was isolated using the magnetic bead method and the silicate column method for bristles and insemination doses, respectively. DNA from the blood samples was isolated using the GeneAll Exgene DNA micro isolation kit (GeneAll Biotechnology, Seoul, Korea). Selected DNA isolates were applied to PorcineSNP60 v2 BeadChips (Illumina, San Diego, CA, USA) using the Illumina HD Infinium technology protocol. A total of 61 565 SNPs were identified in the selected DNA isolates. The average call rate was 0.99, 0.97, and 0.94 for the bristles, blood samples, and insemination doses, respectively. The animals with call rate > 0.90 were used in the subsequent analyses.

Only the SNPs mapped on autosomes were considered in this study. The PLINK v. 1.9 software was used for quality control and subsequent analyses [24]. The SNPs with call rate > 0.90, *p*-value of Hardy–Weinberg equilibrium > 0.0001, and minor allele frequency (MAF) > 0.05 were included in the SNP analyses. After the quality control analysis of all SNPs, the data from 174 animals containing 39 779 SNPs were used for further analysis. Runs of homozygosity (ROH) segments were expressed by a specific minimum number of continuous homozygous SNP markers with a maximum spacing of 1 Mb and a minimum density of one SNP marker per 100 kb. The minimum number of SNPs in ROH (*n* = 39) was determined based on the approach proposed in the study by Lencz et al. to avoid the detection of false-positive ROH segments (α = 0.05) [25]. As shown in previous studies, the length of ROH segments is related to the proportion of genomic information coming from different ancestral generations [26,27,28,29]. Therefore, based on the terminology proposed previously, the ROH segments were divided into five classes on the basis of their length (>1 Mb, >2 Mb, >4 Mb, >8 Mb, and >16 Mb) [27,29]. These trace back approximately 50, 25, 12, 6, and 3 ancestral generations.

The inbreeding coefficients, calculated based on the SNP data, were calculated by four different methods. The genomic inbreeding coefficient was calculated for each ROH (F_ROH_) class by dividing the sum of the ROH segments length (kb) in a particular class by the total length of the autosomal genome (kb) covered by the SNPs and expressed in percentage. The inbreeding coefficient, F_hat1_ is the variance-standardized relationship minus one. The inbreeding coefficient, F_hat2_ is approximately equal to the estimates (observed and expected autosomal homozygous genotype counts for each sample and reported method of moments F coefficient estimates). The inbreeding coefficient F_hat3_ is based on the correlation between uniting gametes. All the inbreeding coefficients were calculated using PLINK 1.9 software [24]. The correlations between evaluated inbreeding coefficients were calculated. The effective population size based on the genomic data was calculated by GONE software [30]. The method is based on the linkage disequilibrium (LD) between SNP markers and implements a genetic algorithm published previously [31]. The parameters were set to 2000 generations for which the linkage data is obtained in bins. Only pairs of SNPs with recombination fraction, c > 1/4000 were considered. Numbers of bins were set to 400, making a five-year generations gap, obtained by dividing the number of generations with the number of bins. However, the first 10 generations were analyzed using 2-generation gaps and the rest using 5-generation gaps. The N_e_ was calculated separately for K1, K2, K3, K4, and K8, which represent 1, 2, 4, and 8 centiMorgans per mega base, respectively. The overall degree of population stratification was assessed by calculating Nei’s genetic distances (Da) between animals and the pair-wise Wright’s index at the group level using the StAMPP R package [32].

## 3. Results

### 3.1. Quality of the Pedigree

**Figure 2:**
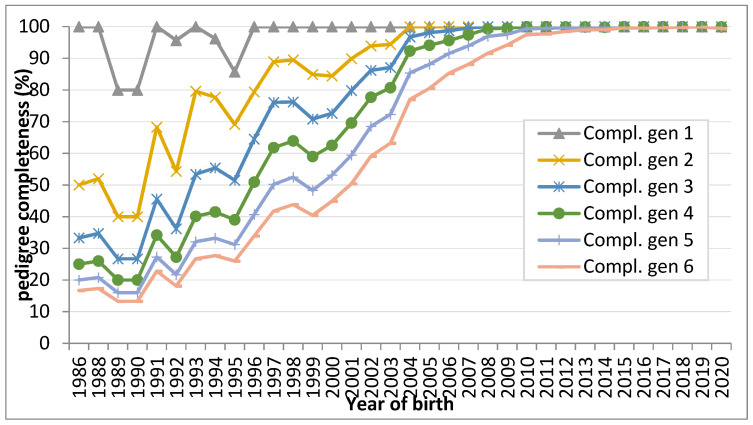
Average pedigree completeness for generation one to six.

**Figure 3:**
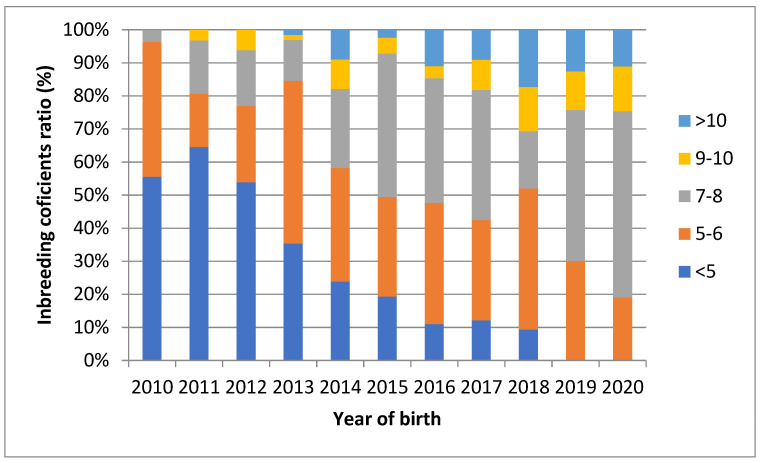
Inbreeding coefficients ratio of animals born between 2010 and 2020. Note: Animals are divided into five groups based on the F_PED_ value. Groups are specified in the figure legend.

**Figure 4:**
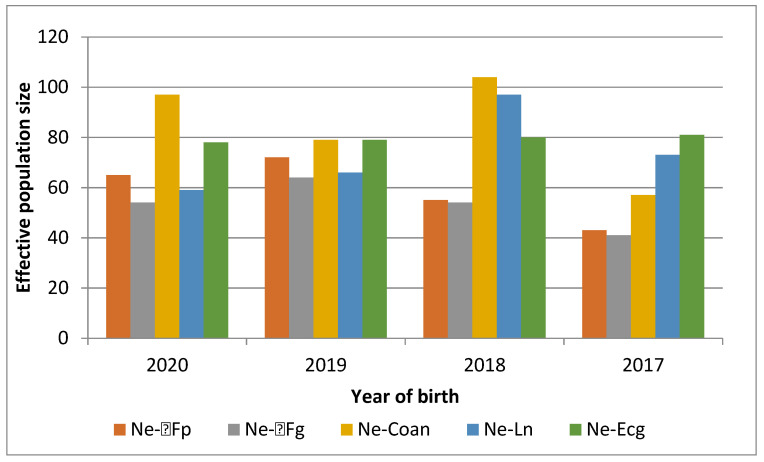
Effective population size (N_e_) based on pedigree data calculated by five methods (described in detail in the Materials and Methods section).

Results from the pedigree data evaluation are presented in Figure 2, Figure 3 and Figure 4 and Table 2, Table 3 and Table 4. The pedigree of the animals in the reference population could be traced back 28 generations at maximum and 12.99 generations on average. The mean value of complete generations was 4.58. For all animals involved in pedigree analysis, the mean equivalent generations were 7.53 and the total number of founders was 269. The number of founders explaining 50% of the diversity was 15 and 7 in animals of pedigree analysis and reference population, respectively.

The PCI calculated for one to six generations back is shown in Figure 2. A PCI value of 100% was observed for all the analyzed generations from 2012. Moreover, a continuous increase in the PCI can be seen from the year 1999 for all generations.

An overview of the GI of animals in the pedigree analysis is presented in Table 2. The average GI for the selection path from sire to son was 3.0 during the study period. This value is the average sire age of 255 selected sons born in the last 30 years. Additionally, the average GI for other selection paths were 2.2 (for both dam to son and dam to daughter) and 2.8 years (for sire to daughter). The average age of parent sires, dams, and overall population was slightly increased by 0.6, 0.2, and 0.4 years in the last 6 years, respectively. This indicates the increased age of parents, especially of sire animals.

### 3.2. Analysis Based on the Pedigree Data

The total inbreeding (F_PED_) observed in the animals from the completely evaluated pedigree was 3.44% on average. Although this value is high, more in-depth analysis is required for a closed population of a genetic resource. Average inbreeding coefficient calculated from the pedigree data for animals born in 2020 was 8.10 ± 1.50%, ranking from 5.50% to 13.00% (see Table 3). The data presented in Table 3 also indicates a gradual increase in the F value in animals born within last three years and also in their parents. There was a slight decrease in the F_PED_ of sires born in 2020. This could be due to the use of boars with less inbreeding. This intervention had only a slight effect on the average F value of their offspring due to the high F value of their mothers used in that particular year.

The overall negative trend of F_PED_ is also observed in Figure 3. The figure shows the proportion of animals with inbreeding divided into groups over the last 10 years. The share of animals with the lowest F_PED_ has been continuously decreasing since 2013. A similar trend was observed for F_PED_ in the 5–6% group. In contrast, there is a continuous increase in F_PED_ in 7–8% group, which in the following generations will cascade leading to an increase in F with a higher proportion. From the context of population development in the future, the rate of the inbreeding (∆F) is an important parameter and is expressed as the difference of the measured inbreeding between two consecutive generations. This value increased at a rate of +1.58% per generation in average. As expected, the inbreeding rate, as calculated by balancing the founder’s contribution, was positive, and was 0.43% per generation.

Table 4 shows the total, new, and old inbreeding calculated for animals born over three recent years (2018–2020) and for their sires and dams. A relatively higher share of the total inbreeding of an individual is coming from the older generations. The ratio F_AnN_ vs. F_AnO_ in animals born in the last three years was 1:2.44. The total inbreeding observed in mothers had a slightly higher share in an individual’s inbreeding than fathers (the ratio of total F between dams and sires was 49:51). However, in a more detailed investigation, we observed differences during the acquisition of inbreeding.

The N_e_ was derived based on multiple parameters depending on various conditions of population under consideration. Different methods also take into account different time windows of pedigree data in calculations and therefore respond differently to rapid changes in population size with different sensitivity. The calculated N_e_ by five methods is compared in Figure 4.

### 3.3. SNP Data Analysis

**Figure 5:**
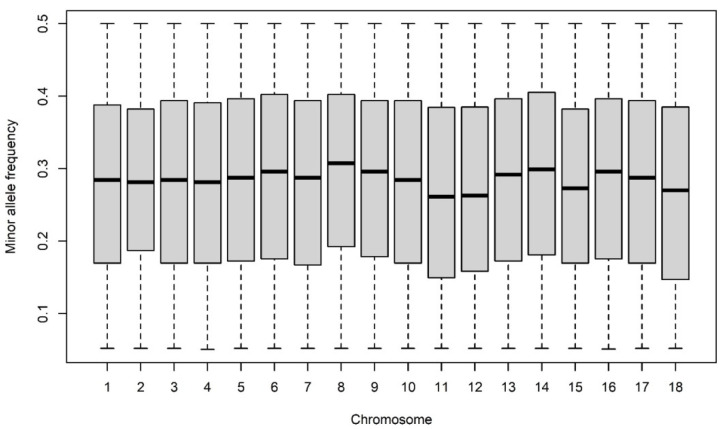
Minor allele frequency (MAF) for each autosome.

The MAF distributed over each autosome are summarized in Figure 5. The average MAF was 0.28 ± 0.11 and ranked from 0.05 to 0.50. There were more than 28% of SNPs with MAF higher than 0.40, whereas less than 10% of SNPs have an MAF of lower than 0.10 in all chromosomes. In total, 2188 SNPs on chromosome eight showed highest average MAF (0.30). In contrast, 1441 SNPs on chromosome six showed the lowest MAF (0.27). The observed (H_o_) and expected (H_e_) within breed heterozygosity was 0.38 ± 0.13 and 0.37 ± 0.12, respectively.

The inbreeding coefficients (F), calculated based on the SNP data, were analyzed by different methods. Table 5 summarizes the results for ROH, namely the number of ROH and F_ROH_ for all considered ROH classes. The F value based on ROH decreased with the increase in the minimum length of ROH. The highest average value for F_ROH_ was observed in class 1 (10.81, varied from 3.23 to 23.66), while the lowest average value for F_ROH_ was in class 16 (3.98, varied from 0.00 to 14.80).

The average value of Fhat1 was −0.026 ± 0.06 and varied from −0.131 to 0.109. The inbreeding coefficient expressed as Fhat2 showed a lower average value compared to that of Fhat1 (−0.032 ± 0.05 and ranged from −0.116 to 0.104). The correlation coefficient between uniting gametes, Fhat3 showed an average value of −0.028 ± 0.046 and varied between −0.086 and 0.107). Sows showed lower Fhat1 values compared to boars (on average, −0.051 and −0.017, respectively). In contrast, an opposite trend was observed for Fhat2 (−0.012 and −0.038 for sows and boars, respectively).

The correlation coefficients between inbreeding coefficients, as determined based on the pedigree and SNP data, are shown in Table 6. The presented coefficients values were highly statistically significant (*p* < 0.001) except for the correlation between F_PED_ and F_hat1_. F_PED_ had a moderate correlation with inbreeding coefficients based on ROH and varied from 0.491 (with F_ROH_ in ROH class 16) to 0.542 (with F_ROH_ in ROH class 8). The correlation observed between F_PED_ and F_hat2_ was also moderate (0.508). However, no relationship was observed between F_PED_ F_hat1_.

**Figure 6:**
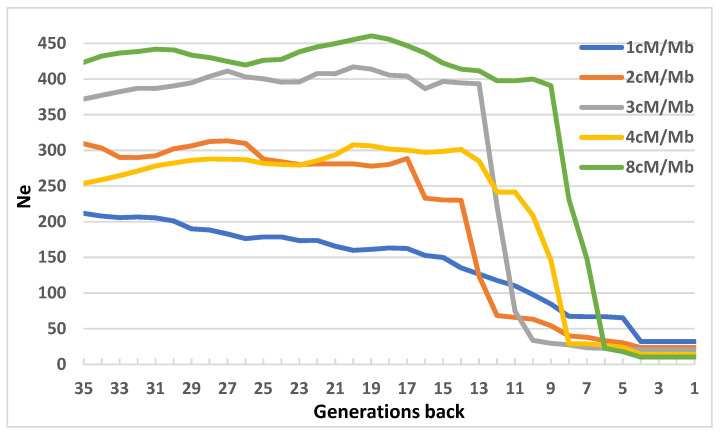
Effective population size derived from the single nucleotide polymorphism data.

The genetic structure all of the individuals in which the SNP data was derived from the Nei’s genetic distance matrix is presented in Supplementary Figure S1. The temporal effective population size (N_e_) calculated using different constant recombination rates is displayed in Figure 6. The presented results show N_e_ up to 35 past generations. In particular, for 1 cM per Mb, the N_e_ value has decreased slowly and could be observed from 8 to 35 past generations. Then, a short period of three generations without any change in N_e_ was observed, followed by a relatively rapid decrease of N_e_ between the fifth to fourth generation. Similar trends were observed for other constant recombination rates with relatively stable historical period from 9 or 13 to 35 past generations, followed by a rapid decrease in N_e_.

## 4. Discussion

### 4.1. Analysis Based on Pedigree Data

Based on the established performance testing and recording of the local PBP breed, the results from the quality of the pedigree are similar to our previous study on the PBP breed [9]. Nevertheless, continued increase of PCI can be seen from 1999 in all generations of pigs under study. For most of the commercial breeds (such as the Czech large white dam and sire line, Czech landrace, and Pietrain) used in the national breeding program called CzePig [16], the completeness of pedigree for more than 10 years was determined only for the first generation of animals. In contrast, slow reduction of PCI recorded in the commercial population at the beginning of the early 1990s also can be seen in the PBP breed. It indicates that, irrespective of the genotype, a breed could be slightly influenced by the import of animals that were unrelated to the PBP breed and/or with the unknown pedigree. Generally, pedigree data, analyzed in present study forms a very good basis for accurate and reliable calculations of basic and derived parameters of genetic diversity of the PBP population.

The GI of the PBP breed (2.5 years on average, see Table 2) is relatively long compared to that in commercial breeds (ranking from 1.40 to 1.95 years), as observed previously [16,33,34]. In contrast, comparable values of average parent age (2.24 to 2.69 years) were measured in various local pig breeds [35,36]. The differences of GI found in our study within the individual selection paths indicate the application of divergent selection pressure. In the context of basic production parameters of the breed (given in the Supplement Table S1), the average number of litters per year is relatively low (1.54 on average), even though the farrowing interval was short (157 days). This corresponds to a longer suckling period of piglets confirmed by the PBP breed farmers (personal communication). In brief, the levels of all meat production and reproduction parameters ratifies the almost extensive production system of this breed.

The average F_PED_ values and average rate of inbreeding (∆F) presented in our study are higher than these values published in the literature. The slightly higher value of F was observed in a previous study for Guadyerbas pig breed maintained in small isolated herds [37]. They reported a value of 3.90%, which is moderately higher than that described in a previous study by Toro et al. on the same breed [38]. We obtained a similar value for F_PED_ in our previous study [9]. However, ∆F observed in the present study is higher. Kock et al. studied the effect of total and partial inbreeding of pigs in Austria; they reported a low correlation (under 0.30) between old and new inbreeding [39]. A study by Silio et al., which analyzed inbreeding coefficients for Iberian pig, reported slightly higher values for inbreeding from the last five generations (57% and 43% contribution of new and old inbreeding, respectively) [40]. In the present study, we reported 71% old inbreeding (Table 4).

It is generally recommended to perform a detailed analysis of the observed N_e_ before making conclusions regarding the genetic diversity of populations [20]. In our study, the N_e_ value of the PBP breed calculated by various methods (described in Materials and Methods section) ranged from 40 to 80 individuals during the years of study (see Figure 3). Similar values were reported in our previous study [9]. In an ideal situation, when population is mating randomly, the values of N_e_-Fg and N_e_-Coan should be the same or very similar to each other. The population is exposed to a certain selection pressure, when N_e_-Coan reaches a higher value than N_e_-Fg. The most appropriate method could be marked N_e_-ecg (as a most stable method with negligible variations over years) or N_e_-ln, which uses shortest time window. Some authors made suggestions for minimal value of N_e_. Welsh et al. stated 50 individuals as the minimum value of N_e_ [41]. Meuwissen and Woolliams recommended that the N_e_ value be maintained between 31 and 250 animals for good population fitness [42]. Tang et al. reported an effective population size of 50 as a threshold necessary to avoid the negative impact of inbreeding in population; these authors recommend that at least 500 animals are needed for maintenance of genetic diversity in population for several generations [34].

### 4.2. SNP Data Analysis

Over the last two decades, enormous progress has been made in using of the SNP data for assessment of genetic diversity. At present, high-density SNPs can be used to analyze the past and current population demography [43]. The MAF observed in the study is relatively high compared to other studies. Muñoz et al. analyzed twenty-one different pig breeds across Europe [8]. They reported relatively moderate differences in MAF (average of 0.228, approximately). Similarly, the results reported in our study suggested that the explanatory power of the SNPs is slightly lower; this could be justified by the fact that local indigenous breeds have not been considered for design of commercial porcine chip. This could be indicated by the results of Hlongwane et al., who reported the genetic variation and population distinctiveness of three commercial, four village, two indigenous, three wild, and one Asian pig breeds [44].

In general, observed and expected heterozygosity of the PBP population are comparable to other studies performed on wide range of pig breeds and they are on the lower range of values published in previous literature [43,45,46]. The observed inbreeding coefficients from the pedigree data (F_PED_) and SNP data were different. It should also be mentioned that the animals in which SNP analysis was preformed represent a wide range of boars and sows born during 2005 to 2020. Even though, all of them are parents of animals used in next breeding, they were not equal to the number of animals in reference populations (only 58% were born in last four years). Schiavo et al. reported a similar trend; they analyzed three commercial and four autochthonous pig breeds raised in Italy for calculation of inbreeding coefficient values and correlation coefficients among the evaluated types of inbreeding [29]. In accordance with their results, in our study the correlations between F_PED_ and F_ROH_ did not vary significantly depending on the changes in ROH length. In contrast, few authors reported a negligible correlation between F_ROH_ and F_PED_ [37]. Zanella et al. reported that this correlation was low in Landrace (0.24) and negligible in Large White (0.015) breed raised in Brazil. The differences in inbreeding coefficients obtained from the pedigree and SNP data could be caused by unknown level of inbreeding in the founder animals in a pedigree [47]. It is assumed, that these founder animals are unrelated with no inbreeding. Additionally, Daetwyler et al. suggested the fact that genomic information estimates the Mendelian sampling effects more accurately, which consequently improves the estimates of the inbreeding rate [48].

From the perspective of current and past effective population size, the population analyzed in our study required an additional attention. In our study, the overall trend in N_e_ is slightly negative, especially over the last three years. Reduction of the number of effective animals recorded in the analyzed breed population has been confirmed both by pedigree and LD analysis.

## 5. Conclusions

The present study reports a detailed analysis of the local PBP pig breed based on all available data. From the results, it is evident that the PBP pig population showed a high degree of inbreeding. Consequently, this results in low value of effective population size. It is necessary to make decisions that stabilize the rate of inbreeding in the long-term by using optimal contribution selection (OCS). The specific mating management, which considers OCS, whereby animals with higher genetic diversity will be preferred for breeding, will be applied in the PBP population in cooperation with breeders’ association. Despite a high volume of pedigree data being recorded, the results of the SNP data analysis should be considered for decision-making in animal conservation programs. However, the final decision-making depends on the understanding of the breeders. For some local populations with very limited pedigree information, the evaluation of genomic information is one of the ways to identify inbred individuals and to quantify the degree of increase in homozygosity due to mating of related animals, respectively.

## Figures and Tables

**Table 1:** Basic characteristics of the population in pedigree analysis.

Parameter (Unit)	Sire	Dam	Total
Total number of animals in pedigree	507	1466	1971
Reference population in pedigree	47	277	324
Number of evaluated herds	12	11	12
Average number of: dams in a herd	15.3		
Sires in a herd	2.71		
Number of offspring selected per dam	1.71		
Number of offspring selected per sire	4.50		
Number of sires per dam	1.19		
Number of dams per sire	3.02		

**Table 2:** Generation interval of animals ^1^ in the pedigree analysis during the study period.

Year of Evaluation	Generation Interval (in Years) in Different Paths ^1^				
	SS	SD	DS	DD	Population
2005	3.6	2.4	2.3	2.4	2.6
2010	1.8	2.0	2.3	2.4	2.1
2015	2.3	2.3	1.8	2.1	2.2
2020	3.3	2.8	2.2	2.2	2.6
Mean	3.0	2.5	2.6	2.2	2.5

^1^ Generation interval expresses the average age of animals when their offspring were born in the year of study. Animals are assigned to the four selection paths: SS: sire to son, SD: sires to daughter, DS: dam to son, and DD: dam to daughter.

**Table 3:** Number and average inbreeding coefficients of animals born in last three years and their sires and dams.

Year	Animals		Sires		Dams	
	n ^1^ in Total	F ^2^	n in Total	F	n in Total	F
2018	75	7.49%	26	7.03%	54	6.82%
2019	103	8.11%	24	7.37%	73	7.21%
2020	126	8.10%	32	6.67%	82	7.43%

^1^ number of observations.^2^ average inbreeding coefficient.

**Table 4:** Average new, old, and total inbreeding coefficients of animals born in three recent years (2018–2020) and their sires and dams (%).

	F_AnN_	F_AnO_	F_AnT_	F_SrN_	F_SrO_	F_SrT_	F_DmN_	F_DmO_	F_DmT_
Avg.	2.31	5.64	7.95	2.15	5.49	7.64	2.32	5.65	7.97
s.d.	2.51	0.71	2.61	1.54	0.71	1.79	1.6	2.61	1.83
Min.	0.00	2.87	4.31	0.00	2.87	5.05	0.00	3.14	4.31
Max	9.38	7.86	14.46	5.47	7.26	12.11	9.38	7.86	14.46

F_AnN_: new inbreeding of animals; F_AnO_: old inbreeding of animals; F_AnT_: total inbreeding of animals; F_SrN_: new inbreeding of sires; F_SrO_: old inbreeding of sires; F_SrT_: total inbreeding of sires; F_DmN_: new inbreeding of dams; F_DmO_: old inbreeding of dams; F_DmT_: total inbreeding of dams; Avg.: arithmetic mean; s.d.: standard deviation.

**Table 5:** Inbreeding coefficient derived from runs of homozygosity (ROH).

ROH Class	No. of ROH			F_ROH_ (%)		
	$\bar{x}$ ± SD	Min	Max	$\bar{x}$ ± SD	Min	Max
1 Mb	41.931 ± 8.974	21	71	10.810 ± 3.634	3.225	23.663
2 Mb	38.477 ± 8.725	19	68	10.571 ± 3.637	3.097	23.289
4 Mb	21.190 ± 5.834	8	36	9.308 ± 3.578	2.438	23.486
8 Mb	9.500 ± 3.941	1	21	6.722 ± 3.320	0.333	20.430
16 Mb	3.517 ± 2.190	0	10	3.984 ± 2.758	0	14.801

**Table 6:** Correlations between inbreeding coefficients ^1^.

	F_ROH1_	F_ROH2_	F_ROH4_	F_ROH8_	F_ROH16_	F_hat1_	F_hat2_	F_hat3_
F_PED_	0.519	0.513	0.510	0.542	0.491	−0.026	0.508	0.307
F_ROH1_		0.998	0.967	0.895	0.795	0.222	0.844	0.659
F_ROH2_			0.964	0.889	0.797	0.220	0.838	0.651
F_ROH4_				0.942	0.834	0.262	0.830	0.689
F_ROH8_					0.893	0.288	0.785	0.686
F_ROH16_						0.237	0.716	0.585
F_hat1_							0.032	0.771
F_hat2_								0.606

^1^ Significant at *p* < 0.001 except for the correlation between F_PED_ and F_hat1_. Detailed description of individual inbreeding coefficients is given in Materials and Methods section.

## Data Availability

The derived data supporting this study is available from the corresponding author on reasonable request.

## Supplementary Materials

The following are available online at https://www.mdpi.com/article/10.3390/genes12121972/s1, Table S1: Basic meat production and reproduction characteristics of the Přeštice Black-Pied pig population in 2020. Figure S1: Genetic structure all of the genotyped individuals in which the SNP data was derived from the Nei’s genetic distance matrix7.
