# Supplementary material for: Assessment of the Genetic Diversity of a Local Pig Breed Using Pedigree and SNP Data
Source: Genes (Basel). 2021 Dec 10;12(12):1972. doi: 10.3390/genes12121972 (PMC8702119; doi:10.3390/genes12121972)
Supplement: Supplementary file 1 [file genes-12-01972-s001.zip › genes-1477832-supplementary-done.pdf]

genes

Supplementary material

Supplementary Table S1. Basic meat production and reproduction characteristics of the Přeštice Black-Pied pig population in 2020.

Parameter (unit)	*n*[4]	*x*[5]	*minimum*	*maximum*	*s.d.*[6]
Number of piglets per single sow litter: born in total	722	10.59	2	18	2.73
born alive	722	9.69	2	16	2.55
weaned	722	8.91	2	15	2.38
Farrowing interval (d)	607	157.43	135	337	38.70
Number of litters born per dam	722	3.89	1	17	3.08
Number of litters born per dam per year	722	1.54	1	3	0.61
Weight of live pig[1] (kg)	399	104.71	70	150	17.26
Average daily weight gain[2] (g)	399	0.56	0.40	0.83	0.06
Backfat thickness[1, 3] (mm)	399	9.18	5	18	1.63
Lean meat content[1, 3] (%)	399	54.34	44.4	63.6	3.03

[1] Recorded in the field at the age of 20–22 weeks.[2] From birth to the end of the field test. [3] Measured sonographically using the Sonomark (however, since 2020 the Mindray has been used). [4] Number of observations. [5] Arithmetic mean. [6] Standard deviation.

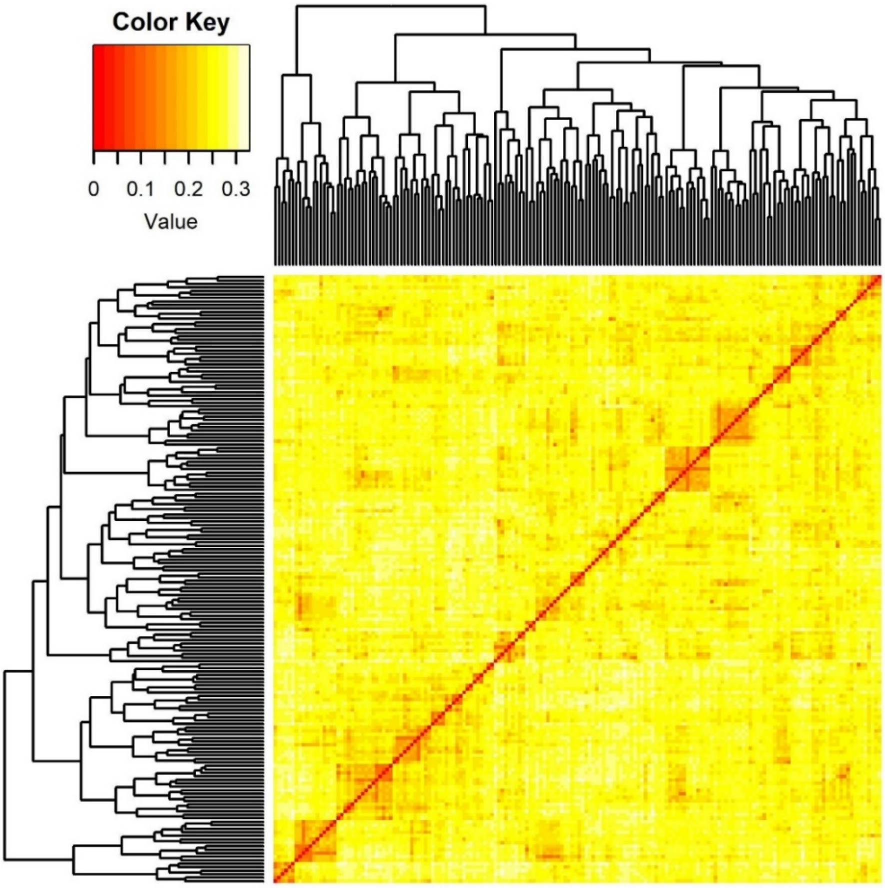

Supplementary Figure S1. Genetic structure all of the genotyped individuals in which the SNP data was derived from the Nei's genetic distance matrix.
